# Herbivore‐induced plant volatiles and tritrophic interactions across spatial scales

**DOI:** 10.1111/nph.14475

**Published:** 2017-02-14

**Authors:** Yavanna Aartsma, Felix J. J. A. Bianchi, Wopke van der Werf, Erik H. Poelman, Marcel Dicke

**Affiliations:** ^1^ Farming Systems Ecology Wageningen University PO Box 430 Wageningen 6700 AK the Netherlands; ^2^ Laboratory of Entomology Wageningen University PO Box 16 Wageningen 6700 AA the Netherlands; ^3^ Centre for Crop Systems Analysis Wageningen University PO Box 430 Wageningen 6700 AK the Netherlands

**Keywords:** herbivore‐induced plant volatiles (HIPVs), host location by parasitoids, landscape ecology, spatial scales, tritrophic interactions, volatile mosaic

## Abstract

Herbivore‐induced plant volatiles (HIPVs) are an important cue used in herbivore location by carnivorous arthropods such as parasitoids. The effects of plant volatiles on parasitoids have been well characterised at small spatial scales, but little research has been done on their effects at larger spatial scales. The spatial matrix of volatiles (‘volatile mosaic’) within which parasitoids locate their hosts is dynamic and heterogeneous. It is shaped by the spatial pattern of HIPV‐emitting plants, the concentration, chemical composition and breakdown of the emitted HIPV blends, and by environmental factors such as wind, turbulence and vegetation that affect transport and mixing of odour plumes. The volatile mosaic may be exploited differentially by different parasitoid species, in relation to species traits such as sensory ability to perceive volatiles and the physical ability to move towards the source. Understanding how HIPVs influence parasitoids at larger spatial scales is crucial for our understanding of tritrophic interactions and sustainable pest management in agriculture. However, there is a large gap in our knowledge on how volatiles influence the process of host location by parasitoids at the landscape scale. Future studies should bridge the gap between the chemical and behavioural ecology of tritrophic interactions and landscape ecology.

## Introduction

Information plays an important role in behavioural choices of individuals, and consequently influences the spatial distribution of populations on larger scales (Vet, 2001; Lof *et al*., 2008; Vinatier *et al*., 2011). Animals have evolved many sensory systems for perceiving cues from their environment, such as vision, hearing, smell and sensing of vibration, and they use a combination of these to make foraging decisions (Roitberg & Gillespie, 2014; Schellhorn *et al*., 2014). In insects, olfaction is the most important sensory system driving behaviour; it influences, among others, food searching, mate finding, avoidance of enemies and competition (Lima & Dill, 1990; Schoonhoven *et al*., 2005). However, little is known about the mechanisms underlying the interactions between insects and their odorous environment in the context of the spatial scales at which these mechanisms need to operate under field conditions.

**Figure 1:**
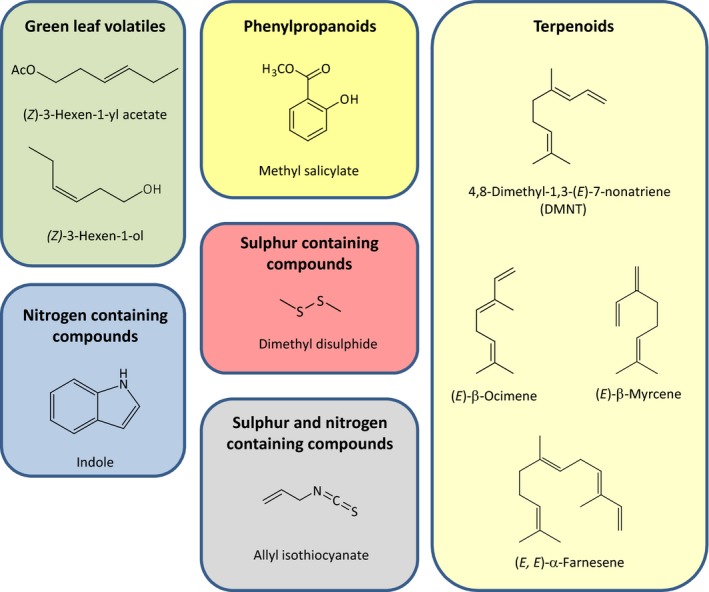
Herbivore‐induced plant volatiles (HIPVs) consist of chemicals from different chemical classes. Examples are provided for several different classes of compounds that can be found in HIPV blends.

Herbivore‐induced plant volatiles (HIPVs) constitute important cues for parasitoids and predators to find prey or hosts (Vet & Dicke, 1992; Hare, 2011). Undamaged plants emit relatively low levels of volatiles. Upon herbivory, plants emit an induced blend of volatiles of different chemical classes (Fig. 1), produced through a variety of biosynthetic pathways. This blend is used by predators and parasitoids as a reliable and well‐detectable cue to find herbivore‐infested plants (Dicke & Baldwin, 2010). While tritrophic interactions mediated by plant volatiles have been extensively studied in the laboratory and small‐scale field experiments (Mumm & Dicke, 2010), many questions remain unanswered about how these interactions unfold beyond the plot/field scale in agroecosystems (James & Price, 2004; Simpson *et al*., 2011).

HIPVs are emitted from plant sources that are heterogeneously distributed at various spatial scales. Individual plants of different species may be induced to different degrees, and by different inducing herbivores, resulting in a complex spatial mosaic of volatile blends. Emitted HIPVs will be transported by wind and turbulence, resulting in mixing of multiple volatile blends, while chemical breakdown will happen at the same time. The compounding of spatially and temporally heterogeneous emission and turbulent transport results in a dynamic and heterogeneous three‐dimensional chemical environment, which we here call the ‘volatile mosaic’. Parasitoids and predators may be able to derive important information from HIPVs within this volatile mosaic, but they may be limited in their ability to detect HIPVs at larger spatial scales due to chemical breakdown of chemical constituents, and mixing of odours from different sources. Furthermore, they may be limited in their ability to initiate directed movement towards these potential sources of hosts/prey, for example if wind speed exceeds the speed of movement. Therefore, the interactions between carnivorous insects and the volatile mosaic are likely to be scale‐dependent, such that different processes may be relevant at different spatial scales.

In this review, we argue that volatile mosaics, by influencing parasitoid choices and, consequently, parasitoid movement, may be a helpful addition to the current suite of landscape ecological concepts, such as structural complexity, fragmentation and connectivity. Volatile mosaics may allow for a more mechanistic understanding of the movement and distribution patterns of organisms that are particularly driven by olfactory cues. Although many carnivorous arthropods use volatile information, we limit our review to interactions between plants, herbivores and primary parasitoids, which lay their eggs in herbivorous hosts. The use of information from their surroundings by primary parasitoids has been extensively studied (van Alphen *et al*., 2003), and their fitness is closely linked to their ability to use volatile information to find hosts (Thiel & Hoffmeister, 2009). We first address the physical characteristics of volatile mosaics and the factors that shape them. Second, we provide information on how parasitoids perceive their environment and how their physical and behavioural traits might influence the extent to which HIPVs are used in a landscape context. Third, we discuss three different spatial scales at which volatile mosaics may influence parasitoid movement and distribution, namely the plant scale, patch scale and landscape scale. Finally, we discuss future research directions, open questions and potential applications of HIPVs for strengthening biological control in agricultural systems.

## Formation of the volatile mosaic and insect behavioural traits

**Figure 2:**
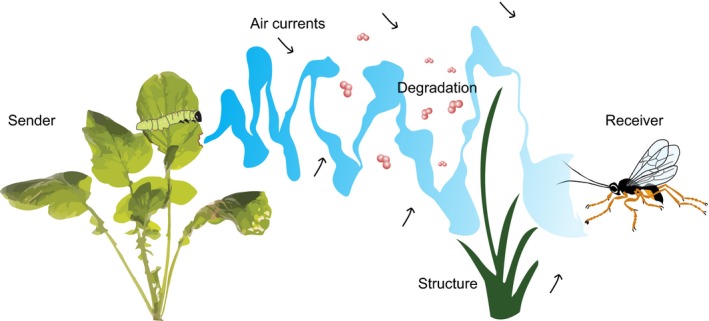
The sender (plant) emits herbivore‐induced volatiles, which disperse as plumes in the environment as a result of air movement. Physical barriers such as vegetation further modulate the movement pattern of the volatile plume. With increasing distance from the source, the plume becomes more fragmented as a result of degradation, by reactions with other compounds in the atmosphere and dilution as airflow spreads the plume. Depending on the distance from the source, and traits of the receiver (insect parasitoid), the receiver may be able to follow the odour plume to the source.

Volatiles emitted by plants form plumes that consist of odour filaments (Murlis *et al*., 1992; Beyaert & Hilker, 2014). These plumes provide information to parasitoids that search for their herbivorous hosts (Dicke & Baldwin, 2010). How this information can be used by parasitoids depends on the sender (the plant), the processes affecting the shape and spatial extent of odour plumes in the environment, and the ability of the receiver (the parasitoid) to perceive the cue (Fig. 2). In this section we discuss these three aspects, of which the first two form the volatile mosaic and the third determines how the volatile mosaic is perceived by the parasitoid.

The production and release of a blend of volatiles starts at the level of the plant. Plant responses to herbivory have been extensively investigated, highlighting that the blend composition may vary with herbivore species, density and herbivore instar (D'Alessandro & Turlings, 2006; Mumm & Dicke, 2010; Rowen & Kaplan, 2016), abiotic conditions (Loreto *et al*., 2014) and plant species, cultivar or even genotype (Degen *et al*., 2004; Poelman *et al*., 2009; Gols *et al*., 2011).

After the odour blend leaves the plant as part of a plume, air currents determine the direction and speed at which the plume travels (Riffell *et al*., 2008). Volatile compounds in the atmosphere can gradually degrade, for example by interactions with reactive chemicals such as ozone (Blande *et al*., 2014). The degradation of compounds can alter the chemical composition of the blend by changing the ratio of compounds within the blend, and/or generating new breakdown products (Šimpraga *et al*., 2016). With increasing distance from the source, a plume becomes more dispersed and probably more difficult to be tracked by parasitoids. These processes ultimately determine the shape, concentration and spatial extent of the odour plume, as well as the composition of the odour filaments, which can alter the information available to insects. In a landscape setting, insects are confronted with assemblages of plants producing odour plumes that differ in blend, strength and size. Limited knowledge is available on the responses of parasitoids to mixed odour plumes (Dicke *et al*., 2003), but mixing of plumes may give rise to complex interactions such as plume masking or plume amplification. For instance, when moths are exposed to a mixture of pheromone and plant volatiles, the capacity of pheromone detection is hampered, probably because of a masking effect of plant volatiles (Deisig *et al*., 2014).

Volatile mosaics consist of assemblages of odour plumes that are scattered across space and can be influenced by the vegetation structure of the landscape. For instance, odour plumes in open fields and forests have different shapes and sizes, possibly due to the differences in wind speed and turbulence in these contrasting habitats (Murlis *et al*., 2000). Increasing plant diversity is expected to increase the structural complexity of vegetation, but can also increase complexity of the volatile mosaic by mixing of odour plumes (Randlkofer *et al*., 2010b). The complex interaction between the spatial arrangement of plant communities in the wider landscape context and environmental factors leads to a bewildering array of emerging patterns, which are likely to change rapidly over time. Yet, parasitoids have to deal with this complexity to obtain olfactory information about the location of their hosts.

The perception of the volatile mosaic by parasitoids is determined by their ability to detect and interpret volatiles. Sensory perception of volatiles by insects relies on olfactory sensilla, primarily located on the antennae. These sensilla are innervated by olfactory receptor neurons, and a wide variety of receptor neuron types can be found among insect taxa (Martin *et al*., 2011; Reinecke & Hilker, 2014). Parasitoid species may differ in their ability to detect volatile compounds, which impacts their ability to discriminate between volatile blends, and the distance from which they can track volatile‐emitting plants (Gouinguené *et al*., 2005). The minimum volatile concentration eliciting a behavioural response may vary between parasitoid species. We expect a positive correlation between sensitivity of a parasitoid species to a particular volatile blend and the distance from which the blend can be detected from the source (as chemical breakdown and dilution due to turbulence have reduced the volatile concentration and altered the composition). Detection of odour plumes in a three‐dimensional environment is complicated because of turbulence and chemical degradation of the plumes over larger distances. Complex navigational strategies are used by insects to locate the source of the odour plume, for instance flying in a zigzagging fashion upwind towards the source of the volatiles (Kaiser *et al*., 1994; Kerguelen & Cardé, 1997; Cardé & Willis, 2008). When following odour plumes, insects may change their navigational strategy at certain distances from the odour source (Willis *et al*., 1991; Bau & Cardé, 2015).

Detection ability is not in itself sufficient to locate a host. Parasitoids should also have the physical ability for directed movement to search and locate the host if they detect HIPVs. We expect flight capacity to influence the scale over which a volatile mosaic is explored and the spatial grain of searching. At low flight capacity, a parasitoid may intensively explore small patches of plants, and may depend on passive dispersal for finding patches further away, while a parasitoid with good capacity for directed flight may visit a sequence of interconnected resource patches by flying upwind in the direction of an odour source. There are several factors influencing the movement capacity of insects. In general, there is a positive correlation between the size of a parasitoid and their movement capacity (Roland & Taylor, 1997). However, even individuals within the same species may exhibit different modes of movement, resulting in displacement across distances ranging from metres to kilometres (Kristensen *et al*., 2013). Host‐specific parasitoids may be more mobile and sensitive to specific volatiles than are parasitoids with a wider host range (van Nouhuys & Ehrnsten, 2004).

## The spatial scales of parasitoid interactions with plant volatiles

A parasitoid female emerging from her cocoon has only limited time to explore the environment and obtain information on patch quality. Perceptual range, resulting from perception sensitivity and odour dispersal, will influence host finding when hosts are heterogeneously distributed. However, not much is known about odour perceptual range of parasitoids in field situations, or whether this range differs between species. Some studies with artificial volatile sources and moths show antennal responses to odour sources in the field up to 60 m from the odour sources, depending on the number of odour sources (Andersson *et al*., 2013). The distance over which odours are perceived also depends on the landscape, which determines how far odours travel. For example, tsetse flies respond to host odours from a much larger distance (60 m) in woodlands than in open fields (20 m), suggesting that odour plumes stay intact longer in these vegetation structures (Voskamp *et al*., 1998). Weather also affects perceptual range by influencing odour plume movement. While plants can convey information on attack by herbivores (Turlings *et al*., 1990; Vet & Dicke, 1992), the detection and interpretation of these cues by parasitoids may differ depending on the distance of the parasitoid to the HIPV source, although empirical evidence for this is lacking (Puente *et al*., 2008). Depending on the spatial scale, different factors may be of overriding importance. Here, we will review the most important factors affecting the response of parasitoids to HIPVs at the plant, patch and landscape scale.

## Plant scale

**Figure 3:**
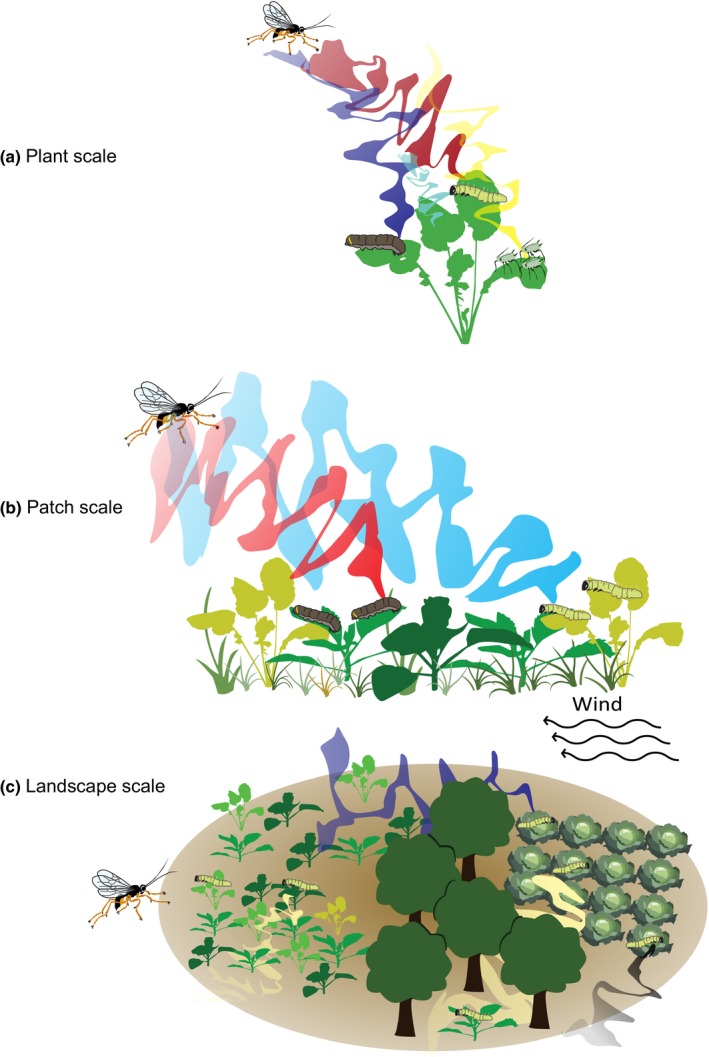
Herbivore‐induced plant volatiles (HIPVs) on multiple spatial scales. (a) A plant can respond to herbivory with the production of HIPVs. The composition of these volatile blends is affected by many on‐plant factors, such as herbivore identity, herbivore feeding guild, herbivore community and plant species or traits. Parasitoid responses may vary with variation in HIPV blends. (b) In nature, the plant is part of a larger community of plants and their associated herbivores. Therefore, parasitoids search for their hosts in patches where a large variety of odour plumes shapes the information on presence of the host, representing a dynamic volatile mosaic. (c) At the landscape scale, different habitats can present different volatile mosaics and distance between these habitats becomes important. Landscape structure as determined by openness of the vegetation and plant diversity, as well as weather conditions such as wind direction, affect how far odour plumes travel. The ability of the parasitoid to perceive HIPVs emanating from patches further away and to move across these habitats influences movement patterns and resulting population distribution.

HIPV release at the plant scale is the basis of the formation of the volatile mosaic, which can be modulated by a wide range of factors, including plant species, plant genotype, plant age, herbivore species, attack severity, abiotic factors or combinations of these (Fig. 3a). The interplay of these biotic and abiotic factors results in specific outcomes of tritrophic interactions at the plant scale in which volatiles may convey reliable information to parasitoids about the infestation of plants by herbivores, while in other cases the volatile cues are less specific (de Rijk *et al*., 2013).

Herbivore‐damaged plants emit a blend that is qualitatively and/or quantitatively different from the blend emitted when the plant is not damaged or mechanically damaged (Turlings *et al*., 1990; Ponzio *et al*., 2014). As a consequence, plants damaged by host herbivores attract more parasitoids than do uninfested or mechanically damaged plants (Turlings *et al*., 1990; Geervliet *et al*., 1994; Potting *et al*., 1995). HIPV emission is positively related to the severity of herbivore damage and herbivore load (but see Shiojiri *et al*., 2010) and, consequently, more heavily infested plants are more attractive to parasitoids (Girling *et al*., 2011). Phloem‐feeding herbivores generally induce lower amounts of volatiles compared with chewing herbivores (Rowen & Kaplan, 2016), possibly because of the limited tissue damage caused by phloem feeders. Besides affecting initial parasitoid attraction to a plant, HIPVs can further stimulate searching behaviour when the parasitoid has arrived on the plant (Uefune *et al*., 2012).

Plant traits can modulate HIPV release and plant volatile emission fluctuates throughout the day (Loughrin *et al*., 1994; Arimura *et al*., 2008), highlighting the dynamic nature of volatile mosaics. Plant species emit specific volatile blends upon attack by the same herbivore species (van den Boom *et al*., 2004). Genotypes or varieties of the same plant species may differ in the intensity of volatile emission (Degen *et al*., 2004; Poelman *et al*., 2009; Gols *et al*., 2011), which may result in contrasting parasitism rates under field conditions (Poelman *et al*., 2009). Additional infestation of the plant by nonhost herbivores may alter HIPV emission and, consequently, parasitoid attraction (de Rijk *et al*., 2013; Chabaane *et al*., 2015; Ponzio *et al*., 2016). Different nonhost‐herbivore species may vary in the degree to which their attack alters HIPV blends and influences parasitoid searching behaviour (Desurmont *et al*., 2014; de Rijk *et al*., 2016). Hence, the contribution of a single plant to the volatile mosaic depends on the attacking insects, both hosts and nonhosts.

## Patch scale

At the patch scale, the complexity of plant communities contributes to the complexity of the volatile mosaic (Fig. 3b). Abiotic conditions, such as wind, influence the distribution of volatiles (Loreto *et al*., 2014; Li *et al*., 2016) and vegetation structure may further modulate the dispersal of volatile blends within the landscape. The variation in HIPV emission between and within plant species is likely to shape volatile blends in complex ways (Degen *et al*., 2004; Poelman *et al*., 2009; Gols *et al*., 2011), which then changes the volatile mosaic depending on the neighbouring plants and the herbivore community that is present on these plants. The effect of plant diversity and habitat complexity on parasitoid behaviour has been extensively studied (Wäschke *et al*., 2014; Kruidhof *et al*., 2015). For example, *Brassica nigra* plants in unmown grassland attracted fewer parasitoids than those in mown grassland or bare soil (Bezemer *et al*., 2010). Parasitoids found host‐infested plants faster in a Brussels sprouts monoculture compared to a Brussels sprout–barley intercrop (Bukovinszky *et al*., 2007). It is unclear whether these differences are caused by volatile masking, mixing of volatiles, or obstruction of visual or olfactory cues by the vegetation. Several effects of background odours on parasitoid behaviour in a patch have been described. If a parasitoid is unable to perceive a certain compound or blend, it is likely that this compound does not alter its behaviour in a patch (Schröder & Hilker, 2008). A background odour might attenuate a behavioural response if it masks the target odour, or enhance the response if it complements the signal of the HIPV‐emitting plant (Schröder & Hilker, 2008).

The induction of HIPVs in neighbouring plants infested with nonhost herbivores can stimulate the searching efficiency of parasitoids by creating a contrasting HIPV blend that can help the parasitoid to identify the HIPV blend of host‐infested plants (Soler *et al*., 2007; de Rijk *et al*., 2013). Yet, this effect may depend on the herbivore species inducing the neighbouring plant. For instance, discriminating hosts from nonhosts on the basis of HIPVs is more difficult for parasitoids when the herbivores are from the same feeding guild than from a different feeding guild (Geervliet *et al*., 1996; de Rijk *et al*., 2013). The attractiveness of neighbouring plants can also influence the searching behaviour of parasitoids on host‐infested plants. When an attractive host plant was surrounded by less attractive, but still attractive nonhost plants, the searching efficiency on the host plant decreased, suggesting that the perception of the patch quality exceeds the scale of a single plant (Perfecto & Vet, 2003). HIPVs can attract parasitoids to host patches, and may even be used to assess patch quality. Aphid parasitoids that were previously exposed to a plant with a high aphid density spent less time on plants with few or no aphids than parasitoids that were previously exposed to a plant with a low aphid density. This was independent of actual presence of aphids during the exposure period, suggesting that plant volatiles were used by the parasitoids to assess patch quality (Tentelier & Fauvergue, 2007).

Theoretical studies with plume models suggest that odour sources of which the plumes can be perceived at larger distances attract more insects than those than those that can be perceived at only a short distance from the source (Manoukis *et al*., 2014). Long‐distance spread of odour plumes facilitated host location if hosts were sparsely distributed, while short‐ or long‐distance spread of plumes equally facilitated host location if hosts were dense (Puente *et al*., 2008). The release of the synthetic HIPV component phenylethyl alcohol influenced the community composition and abundance of a range of arthropod taxa (both second and third trophic level) up to at least 8 m away, both positively (by attraction) and negatively (by repellence) in soybean fields (Braasch & Kaplan, 2012). By contrast, Mallinger *et al*. (2011) found that such effects were more localised and ceased at 1.5 m from the source. These spatial characteristics of the response to odour plumes are likely to be both plant‐ and insect‐species specific.

## Landscape scale

Landscapes are composed of a mosaic of vegetation patches, each consisting of plants that may produce HIPV plumes. However, only a few studies have examined how HIPVs influence the movement of parasitoids at a landscape scale and how HIPVs from different patches influence the distribution of parasitoids across a landscape (James & Price, 2004; Simpson *et al*., 2011). The study of HIPVs in a landscape context presents a challenge because of the difficulty of tracking parasitoid movement at large spatial scales, and because HIPV plumes are not visible and, therefore, hard to assess in the field. Indeed, most landscape‐scale studies infer parasitoid movement via indirect methods, such as analysis of meta‐population structure, rather than by assessing the movement paths of individual parasitoids (Schellhorn *et al*., 2014). Nevertheless, considering HIPV plumes may reveal important insights into the movement and distribution patterns of parasitoids at the landscape scale.

Landscape‐ecological studies have shown that forest edges, proportion semi‐natural area or landscape simplification can have profound impacts on the distribution of parasitoids and their impact on herbivore populations (Pollard & Holland, 2006; Bianchi *et al*., 2008; Woltz *et al*., 2012; Rusch *et al*., 2016), and that parasitoids respond to the landscape context at spatial scales ranging from several hundred metres to kilometres (Thies *et al*., 2003; Bianchi *et al*., 2008). While it has been shown that habitat types and vegetation structures may foster or impede parasitoid movement (Cronin, 2003a,b), it is not clear to what extent HIPVs play a role in this. The volatile mosaic can be considered as an additional information layer on top of the structural vegetation pattern. Depending on the spatial arrangement of vegetation patches emitting HIPVs and meteorological conditions that determine the shape and direction of odour plumes, the volatile mosaic may facilitate parasitoid movement (e.g. when nearby odour plumes function as stepping stones) or interfere with it (e.g. when attractive odour plumes are masked by less attractive plumes), in similar ways as found for vegetation structures (Tscharntke & Brandl, 2004) (Fig. 3c). While the spatial vegetation template varies at a seasonal time scale, the volatile mosaic is much more dynamic and may change within seconds to minutes depending on wind conditions, turbulence and vegetation structure.

Understanding the interactions between parasitoids, herbivores and plants in a volatile‐mosaic context requires the integration of various factors that have been addressed in this review. In a landscape, parasitoids need to find all ecological requisites, including food resources, hosts and mates, and they need to allocate time to finding these resources at appropriate times of their lives (Lewis *et al*., 1998; Landis *et al*., 2000). The perception of the volatile mosaic may be very different depending on the scale and mode of movement of the parasitoid. For instance, volatile mosaics may be perceived as fragmented by parasitoids with a limited mobility, while less so by parasitoids with a large dispersal capacity (van Nouhuys, 2005). Further work is needed to unravel the factors and mechanisms that underlie the parasitoid movement and host searching at the landscape scale.

## Future perspectives

The previous sections show that knowledge of parasitoid responses to HIPVs within the volatile mosaic decreases with increasing spatial scale. At present, there are no accurate data on the spatial extent of HIPV plumes. Beyond a critical distance, it can be expected that HIPV plumes are simply too diluted or dispersed by turbulence or chemically degraded, so that no reliable information can be derived from them by parasitoids. We hypothesise that HIPV plumes may provide reliable cues for parasitoids up to a distance of tens of metres, in line with studies on flies and moths (Voskamp *et al*., 1998; Andersson *et al*., 2013) and parasitoids (Y. Aartsma, pers. obs.). At further distances other cues will have overriding importance. It has been proposed that herbivores, pollinators and parasitoids use general ‘habitat cues’ to find locations that potentially contain resources, and then switch to more specific cues within this habitat (Vinson, 1976; Webster & Cardé, 2016). Indeed, in no‐choice situations or choice situations against nonhost plant species, parasitoids often also respond to volatiles from undamaged plants, indicating that in the absence of host‐specific HIPVs, more general cues are used (Gohole *et al*., 2005; Moraes *et al*., 2008). Hierarchical plume switching is a possible mechanism by which flying insects following a long‐range habitat cue might switch to following more reliable short‐range cues (Beyaert & Hilker, 2014). A better understanding of the functioning of HIPVs in realistic field conditions requires characterisation of the distance over which odour plumes can attract parasitoids in different plant–herbivore combinations.

Assessing the response of parasitoids to volatile mosaics is methodologically challenging, especially at the field scale and beyond. Here, we propose three potential approaches that integrate approaches from chemical ecology and landscape ecology that may foster progress in this field. First, the mechanistic basis of the volatile mosaic can be studied by collecting HIPV blends under field conditions in habitats with different structural complexity. By presenting field‐collected or synthetic HIPV blends to parasitoids and recording their behaviour, predictions for parasitoid behaviour and distribution in the field can be made. Parasitoids can be released at different distances from a source and recaptured near the source to determine the distance at which parasitoids respond in the field (Papaj & Vet, 1990). Furthermore, electro‐antennographic (EAG) measurements in the field can be used to study in more detail under which conditions HIPV blends are still distinguishable against a background (Milli *et al*., 1997; Andersson *et al*., 2013; Misztal, 2016). Second, the volatile mosaic may be studied at a landscape scale by assessing the spatial distribution of parasitoids in landscapes with different numbers and spatial arrangements of HIPV sources. In addition, to assess the effect of an HIPV source in specific volatile‐mosaic contexts, the response of parasitoids could be studied by introducing standardised HIPV sources in different habitat patches. The effect of the strength of HIPV cues could further be assessed in detail by using plant phenotypes that clearly differ in HIPV emissions (Poelman *et al*., 2009). We hypothesise that variation in relative volatile emission rate and associated variation in attraction of parasitoids and predators among plant species/genotypes are strongly dependent on the volatile mosaic in the surrounding landscape. Third, by studying different parasitoid species with well‐known functional traits, such as threshold HIPV concentrations to initiate host‐searching behaviour, important new insights may be acquired about how parasitoid distribution patterns in realistic landscape settings are shaped by the interaction between species traits and the volatile mosaic. We hypothesise that parasitoid species traits such as size and dispersal capacity influence the spatial scale and landscape context at which they respond to HIPVs. Finally, simulation models may be used to integrate and extend information about parasitoid responses to volatile mosaics. For instance, simulations suggest that wind direction and HIPV concentration are important factors determining the spatial distribution of HIPVs (Kuroyanagi *et al*., 2012). Such modelling studies may generate hypotheses that can be experimentally tested in field experiments (Manoukis *et al*., 2014).

Interactions between parasitoids and the volatile mosaic should be studied at relevant spatial scales. These relevant spatial scales may be species‐specific because parasitoids differ in dispersal capacity and search behaviour, and they may also depend on vegetation structure and meteorological conditions. Parasitoids with a low dispersal capacity may lack the ability for directed movement to distant targets and may therefore be less responsive to cues from longer distances, while larger parasitoids with a good capacity for directed search may be more sensitive to long‐range cues. However, for many parasitoids knowledge about functional spatial scales is limited.

Finally, it is important to recognise that volatile cues are not the only information available for parasitoids to find their hosts, and that host searching is only a part of the daily activities. Visual and vibrational cues are also used in host finding (Fischer *et al*., 2001), although these are considered short‐range cues (Völkl, 2000). Moreover, vegetation structure can influence parasitoid movement patterns, not only through the volatile mosaic (Randlkofer *et al*., 2010b), but also by visual obstruction, physical increase of the searching area (Gols *et al*., 2005; Randlkofer *et al*., 2010a), or effects on parasitoid flight capacity. In field situations, insects are likely to use multiple modes of information acquisition (Kulahci *et al*., 2008). Our understanding of the interactions between parasitoids and volatile mosaics can benefit from a better integration of chemical, behavioural and landscape ecological approaches.

## Applications

In natural systems, parasitoids and predators keep populations of herbivorous insects at low levels, and they can have similar impacts in agriculture (Ramsden *et al*., 2016). Attracting natural enemies to crop fields therefore would be beneficial for farmers. There are indications that as a consequence of crop domestication and reliance on insecticides, many crops have reduced defences against herbivore attack and reduced attractiveness to natural enemies compared to their wild relatives (Chen *et al*., 2015). However, a recent meta‐analysis suggests that emission rates of HIPVs, especially green leaf volatiles and sesquiterpenes, are actually higher in crops than in wild plant species (Rowen & Kaplan, 2016). The complexity of volatile blends from domesticated crops is reduced as compared to wild species, which may mean that compounds which are important in attracting natural enemies are limiting (Rowen & Kaplan, 2016). Modern plant breeding mainly focuses on direct mechanisms of resistance, and little attention is paid to the development of improved indirect defence mechanisms, for example through natural enemy attraction by plant volatiles (Ǻhman *et al*., 2010).

Another challenge for the application of HIPVs in pest management strategies is that the reported effectiveness of HIPVs is mixed. Success stories include the ‘push–pull’ system developed for maize production (Khan *et al*., 1997), and the effects of white cabbage varieties that are more attractive to parasitoids in the laboratory in combination with higher parasitism rate in the field (Poelman *et al*., 2009). However, other studies show that parasitoid searching activity and host finding success are increased in laboratory studies, but that these changes do not result in reduced pest populations in the field (Halitschke *et al*., 2008; von Mérey *et al*., 2012; Bruce *et al*., 2015). This illustrates the importance of field studies in addition to detailed behavioural studies in the laboratory.

For biological control, it is important that crops can attract sufficient natural enemies for effective top‐down control of herbivore populations. There have been mixed results with engineering constitutive release of plant volatiles or alarm pheromones (Bruce *et al*., 2015) and simulations show that HIPVs as cues do not increase parasitism rates when plants emit them in the absence of hosts of parasitoids (Kaplan & Lewis, 2015). Enhancing induced crop attractiveness (by triggering a higher HIPV emission upon herbivory) might be a more useful approach to increase attraction of natural enemies within as well as into the crop (Kappers *et al*., 2011). Also, monoculture cropping systems are often very simplified and exposed to frequent disturbances, and they therefore rely on recruitment of natural enemies from the surrounding habitat (Wissinger, 1997). In addition, natural enemies may rely on floral resources that can only be found outside the field (but see Vollhardt *et al*., 2008).

Crops with enhanced HIPV emission levels may reduce natural enemy populations in neighbouring crops (Braasch & Kaplan, 2012). Indeed, parasitoid redistribution on a local scale (8 m) was observed after volatile lures were used, resulting in increased braconid parasitoid densities near the lure, but lower densities in plots further away from the lure (Braasch & Kaplan, 2012). Other arthropod taxa, however, did not show such natural enemy depletion responses, and it is unclear what the consequences will be at larger spatial scales. This suggests that the implementation of strategies to enhance natural enemy recruitment by crops with enhanced HIPV emission must go hand in hand with habitat management to conserve and increase natural enemy populations near crops (Landis *et al*., 2000; Tscharntke *et al*., 2005).

## Conclusions

In the last few decades we have learned a lot about HIPVs in terms of (bio)chemistry, plant physiology and behavioural ecology of insect responses to HIPVs (Turlings *et al*., 1990; Mumm & Dicke, 2010). The focus of this research was particularly on interactions between individual plants and a single herbivore and the response of individual parasitoids at the plant level. In more recent years, research has addressed the effects of HIPVs within a community context, again particularly at the plant level (Desurmont *et al*., 2014; Stam *et al*., 2014). Despite exciting advances in research on responses of plants to herbivory, many important questions remain unanswered about the consequences of HIPV emissions for parasitoid foraging behaviour and host–parasitoid population dynamics in field or landscape settings. These questions, relating to the spatial scale at which HIPVs operate, are crucial for our understanding of tritrophic interactions and possible applications of volatiles in agricultural pest management (Gish *et al*., 2015). Research on HIPVs will need to consider effects at larger spatial scales if it is to assess the effects on populations in a spatial context and contribute to durable pest management in an agricultural context. Current mechanistic understanding of the effect of plant volatiles on insect movement may be used to formulate empirically testable hypotheses on the role of HIPVs in ecological processes at the larger spatial scales that are important for landscape ecology and agricultural pest management.
